# Silencing Core Spliceosome Sm Gene Expression Induces a Cytotoxic Splicing Switch in the Proteasome Subunit Beta 3 mRNA in Non-Small Cell Lung Cancer Cells

**DOI:** 10.3390/ijms21124192

**Published:** 2020-06-12

**Authors:** Maxime Blijlevens, Malgorzata A. Komor, Rocco Sciarrillo, Egbert F. Smit, Remond J. A. Fijneman, Victor W. van Beusechem

**Affiliations:** 1 Amsterdam UMC, Medical Oncology, Cancer Center Amsterdam, Vrije Universiteit Amsterdam, de Boelelaan 1117, 1081 HV Amsterdam, The Netherlands; m.blijlevens@amsterdamumc.nl (M.B.); g.komor@nki.nl (M.A.K.); r.sciarrillo@amsterdamumc.nl (R.S.); 2 Department of Pathology, Netherlands Cancer Institute, Plesmanlaan 121, 1066 CX Amsterdam, The Netherlands; r.fijneman@nki.nl; 3 Amsterdam UMC, Pediatric Oncology and Hematology, Cancer Center Amsterdam, Vrije Universiteit Amsterdam, de Boelelaan 1117, 1081 HV Amsterdam, The Netherlands; 4 Department of Thoracic Oncology, Netherlands Cancer Institute, Plesmanlaan 121, 1066 CX Amsterdam, The Netherlands; e.smit@nki.nl; 5 Amsterdam UMC, Pulmonary Diseases, Vrije Universiteit Amsterdam, de Boelelaan 1117, 1081 HV Amsterdam, The Netherlands

**Keywords:** non-small cell lung cancer, alternative splicing, proteasome, Sm proteins

## Abstract

The core spliceosomal Sm proteins were recently proposed as cancer-selective lethal targets in non-small cell lung cancer (NSCLC). In contrast, the loss of the commonly mutated cancer target SF3B1 appeared to be toxic to non-malignant cells as well. In the current study, the transcriptomes of A549 NSCLC cells, in which SF3B1 or SNRPD3 was silenced, were compared using RNA sequencing. The skipping of exon 4 of the proteasomal subunit beta type-3 (PSMB3) mRNA, resulting in a shorter PSMB3-S variant, occurred only after silencing SNRPD3. This observation was extended to the other six Sm genes. Remarkably, the alternative splicing of PSMB3 mRNA upon Sm gene silencing was not observed in non-malignant IMR-90 lung fibroblasts. Furthermore, PSMB3 was found to be overexpressed in NSCLC clinical samples and PSMB3 expression correlated with Sm gene expression. Moreover, a high PSMB3 expression corresponds to worse survival in patients with lung adenocarcinomas. Finally, silencing the canonical full-length PSMB3-L, but not the shorter PSMB3-S variant, was cytotoxic and was accompanied by a decrease in proteasomal activity. Together, silencing Sm genes, but not SF3B1, causes a cytotoxic alternative splicing switch in the PSMB3 mRNA in NSCLC cells only.

## 1. Introduction

The human genome consists of approximately 20,000 protein-coding genes, but the transcriptome and proteome are considerably larger. This is brought about by, amongst other processes, alternative RNA splicing. RNA splicing converts premature mRNA into mature protein-coding mRNA through the removal of intronic sequences. Different mRNA transcripts can be formed through alternative splicing processes, including alternative 3′ or 5′ splice site selection, exon skipping or intron retention [1]. Different mRNA splice variants encode different protein isoforms, which can have distinct, or even opposing functions. Alternative splicing (AS) is a common process in healthy cells, occurring in up to 94% of human genes [2]; and is crucial for proper tissue and organ development [3]. However, when RNA splicing becomes deregulated, this can contribute to tumorigenesis through the loss-of-function of tumor suppressor genes or the gain-of-function of oncogenes [4]. Over recent years, efforts have been made in exploiting the RNA splicing machinery as a therapeutic target in cancer. The chosen strategies and reported effects have been reviewed extensively [5,6]. RNA splicing is a dynamic reaction catalyzed by a ribonucleoprotein complex called the spliceosome, involving the sequential recruitment and release of many different proteins to and from the pre-mRNA transcript. Aside from serine/arginine-rich protein-specific kinases, by far the most abundantly studied splicing factor as a target for cancer therapy is splicing factor 3B subunit 1 (SF3B1), which is frequently mutated in cancer, especially in hematological malignancies [7]. Small molecules targeting this splicing factor have been developed and are being tested in clinical trials, with varying outcomes [8,9].

We recently found that another class of splicing factors, i.e., the Sm proteins that form a ring structure in the core of the spliceosome, are cancer-selective lethal targets. The silencing of any of the seven Sm genes killed non-small cell lung cancer (NSCLC) cells, but not non-malignant cells. In contrast, targeting SF3B1 was toxic to malignant, as well as non-malignant lung cells [10]. Thus, the Sm proteins appeared to be particularly attractive targets to combat NSCLC.

To date, it is unknown if and what changes in RNA splicing patterns are associated with loss of NSCLC cell viability in response to Sm gene silencing, and if these events are distinct from those induced by silencing SF3B1. Therefore, in the current study, we compared the transcriptomes of NSCLC cells before and after silencing an Sm gene or SF3B1 by using RNA next-generation sequencing. From this analysis, we found an alternative splicing switch in the proteasomal subunit beta type-3 (PSMB3) mRNA, from the canonical long variant (PSMB3-L) to a short variant (PSMB3-S) that is predicted to be targeted for nonsense-mediated decay (NMD). This switch occurred in NSCLC cells when any of the seven Sm genes were silenced, but not when SF3B1 was silenced or inhibited with the splicing modulator pladienolide B. The observed cytotoxicity could be attributed to a loss of PSMB3-L. We conclude that Sm gene knockdown, but not SF3B1 knockdown, causes a cytotoxic alternative splicing switch in the PSMB3 gene in NSCLC cells, resulting in a loss of the full length protein. This switch is likely to contribute to the cancer-selective toxicity of silencing Sm genes.

## 2. Results

### 2.1. Analysis of Transcriptomic Changes in NSCLC Cells after Silencing SNRPD3 or SF3B1

To investigate transcriptomic changes in NSCLC cells in response to splicing factor silencing, an RNA sequencing experiment was performed on A549 NSCLC cells transfected with a non-targeting control siRNA (siNT), an siRNA targeting SNRPD3 (siSNRPD3), or an siRNA targeting SF3B1 (siSF3B1). A schematic overview of the experimental set-up is given in Figure S1.

**Figure 1:**
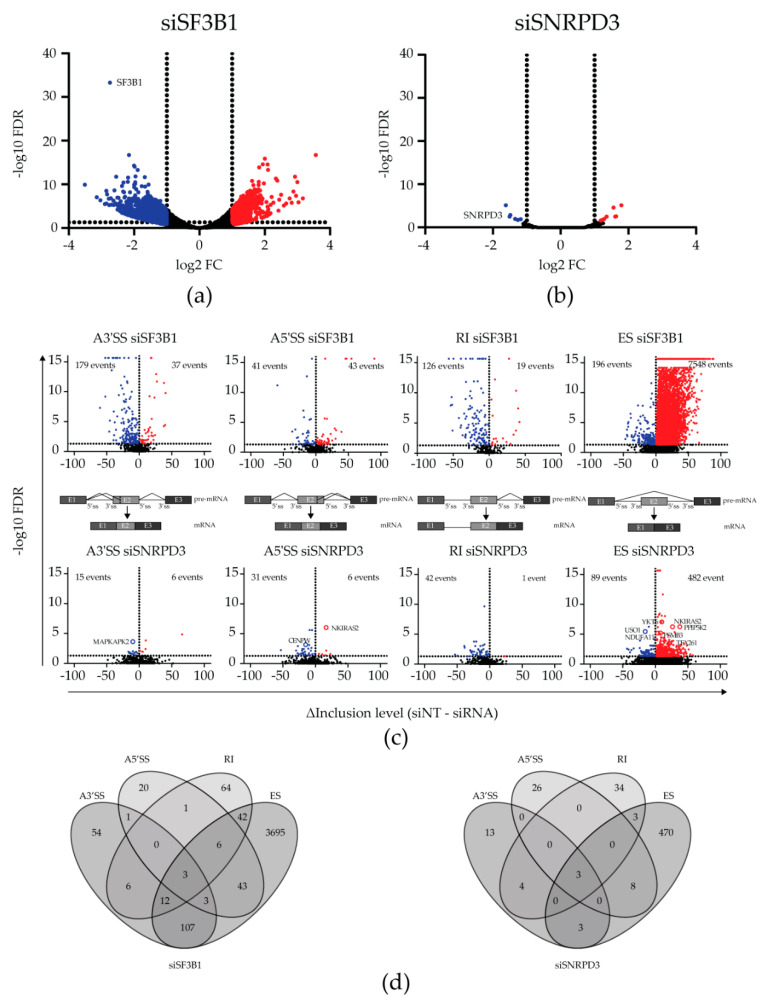
Transcriptomic changes upon silencing SNRPD3 compared to silencing SF3B1. (**a**) Differential expression (DE) analysis revealed 729 upregulated and 1260 downregulated genes when SF3B1 was silenced in A549 non-small cell lung cancer (NSCLC) cells (FDR < 0.05, logFC < −1 or > 1). (**b**) In contrast to siSF3B1, only eight and six genes were up- or downregulated upon SNRPD3 knockdown, respectively (FDR < 0.05, logFC < −1 or > 1). (**c**) Alternative splicing (AS) events (A3′SS, A5′SS, RI and ES) upon silencing SF3B1 (top) or SNRPD3 (bottom). Silencing SF3B1 led to considerably more AS events than silencing SNRPD3. (**d**) Venn diagrams showing the overlap between the types of AS events within samples. Upon SF3B1 silencing, multiple AS events occurred within the same gene, whereas SNRPD3 silencing led to mostly single AS events within genes.

As expected, a highly significant downregulation of SF3B1 and SNRPD3 mRNA was observed in siSF3B1 and siSNRPD3 treated cells, respectively, confirming effective gene silencing (Figure 1a,b). Silencing SF3B1 resulted in the differential expression (DE) of 1988 genes (false discovery rate (FDR) < 0.05; log fold change > 1 or < −1), of which 729 genes were upregulated and 1259 genes were downregulated (Figure 1a; Table S1). In contrast, SNRPD3 knockdown led to the DE of only 13 genes other than SNRPD3 (of which eight were upregulated and five were downregulated; Figure 1b; Table S2). Thus, silencing SF3B1 had much more profound effects on the transcriptome than silencing SNRPD3. Gene ontology (GO) term analysis using the DAVID functional annotation tool [11,12] revealed a significant enrichment of genes involved in transcriptional regulation and DNA replication in the siSF3B1 upregulated and downregulated gene set, respectively (Table S1). The number of differentially expressed genes upon SNRPD3 knockdown was too low to perform GO term enrichment analysis (gene annotation is shown in Table S2). Only five differentially expressed genes (i.e., ATF3, DUSP5, TM4SF1, GSTP1 and UBE2L6) were shared between siSF3B1- and siSNRPD3-transfected cells. Hence, the detected effects of silencing SF3B1 or SNRPD3 on the transcriptome of A549 NSCLC cells were largely different.

Next, the alternative splicing of RNA transcripts in response to splicing factor silencing was analyzed using the rMATS algorithm [13]. Here, the transcriptomes of siSF3B1-treated cells and siSNRPD3-treated cells were each compared to the transcriptome of siNT-transfected control cells. Volcano plots of filtered AS events are displayed in Figure 1c. For siSF3B1, 8189 AS events in 4057 unique genes were detected (FDR < 0.05), of which 95% were exon skipping events. Thus, multiple AS events were often detected in the same gene. The analysis of individual events revealed that, while multiple exon skipping events often occurred in the same gene, alternative splice site usage and intron retention usually occurred only once within the same gene and were largely mutually exclusive. These events did, however, regularly co-occur with an exon skipping event in the same gene (Figure 1d).

When SNRPD3 was silenced, only 672 AS events (FDR < 0.05, Figure 1c) were observed, of which most were exon skipping events. However, in contrast to what we observed upon the silencing of SF3B1, the knockdown of SNRPD3 caused mainly single AS events in individual genes (96%; Figure 1d). Therefore, overall, silencing SNRPD3 appeared to cause more distinct changes in the transcriptome of A549 cells than silencing SF3B1, with fewer mRNAs affected and fewer AS events per mRNA.

### 2.2. Identification and Validation of AS Events Unique to the Knockdown of SNRPD3 Compared to SF3B1

**Figure 2:**
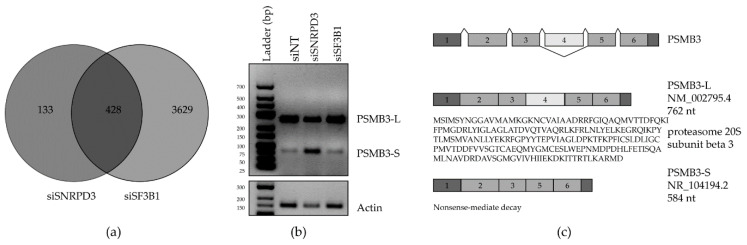
Identification and validation of alternative splicing (AS) events unique to the knockdown of SNRPD3 compared to SF3B1. (**a**) Venn diagram showing the overlap in AS events identified in siSF3B1- and siSNRPD3-treated cells. (**b**) AS of the proteasomal subunit beta 3 (PSMB3) was confirmed by PCR to occur only upon SNRPD3 silencing. Silencing SNRPD3 in A549 cells led to the decreased expression of the long splice variant (PSMB3-L) and the increased expression of the short variant (PSMB3-S). (**c**) PSMB3-L is the canonical full-length transcript variant that is translated into protein, whereas the truncated PSMB3-S variant is predicted to be targeted for nonsense-mediated decay, and is therefore non-coding.

As we identified Sm proteins, but not SF3B1, as cancer-selective lethal targets in NSCLC, we sought to identify AS events that occur in NSCLC cells exclusively upon Sm silencing compared to SF3B1. To this end, we compared all unique genes with AS events in siSF3B1- and siSNRPD3-transfected A549 cells (Figure 2a). GO term analysis on unique siSNRPD3 AS genes (Table S3), unique siSF3B1 AS genes (Table S4) or AS genes in both sets (Table S5) revealed the enrichment of genes involved in RNA splicing. Hence, intriguingly, it appears that interfering with SF3B1 and/or SNRPD3 expression induced the AS of many other splicing factor genes.

The complete list of AS events in the 133 mRNAs in the siSNRPD3-specific gene set is given in Table S3. The top 10 most significant AS events in this set, occurring in nine different genes, are depicted in the volcano plots for siSNRPD3 in Figure 1c and were selected for validation by either quantitative RT-PCR or endpoint PCR using AS-specific primers (Figure S3 and Table S6). For some mRNAs, AS could not be confirmed, or was inconclusive due to the presence of multiple splice variants. The most convincing AS events in this analysis were the exon skipping events in TEX261, YKT6 and PSMB3. For TEX261, as identified by the rMATS analysis, silencing SNRPD3, but not SF3B1, resulted in the skipping of exon 3. For YKT6, silencing SNRPD3 led to a decrease in the canonical full-length variant and an increase in the shorter variant in which exon 6 is skipped. This was also observed upon SF3B1 silencing, but to a lesser extent. Additionally, a product of about 300 nucleotides appeared in siSNRPD3-treated cells only. There is no described or predicted YKT6 splice variant that could explain this observation. The most prominent difference between siSNRPD3- and siSF3B1-transfected cells was the exon 4 skipping event in the proteasome subunit beta 3 (PSMB3) gene (Figure 2b). Therefore, we focused our attention on this specific event. The skipping of PSMB3 exon 4 results in a 178 nt shorter transcript variant (PSMB3-S, Figure 2c), which is predicted to be a target for nonsense-mediated decay (NMD). Hence, exon 4 skipping is expected to result in decreased PSMB3 protein expression.

### 2.3. Sm Gene Silencing Induces a Cytotoxic Alternative Splicing Switch in PSMB3 that is More Pronounced in NSCLC Cells than in Non-Malignant Cells

**Figure 3:**
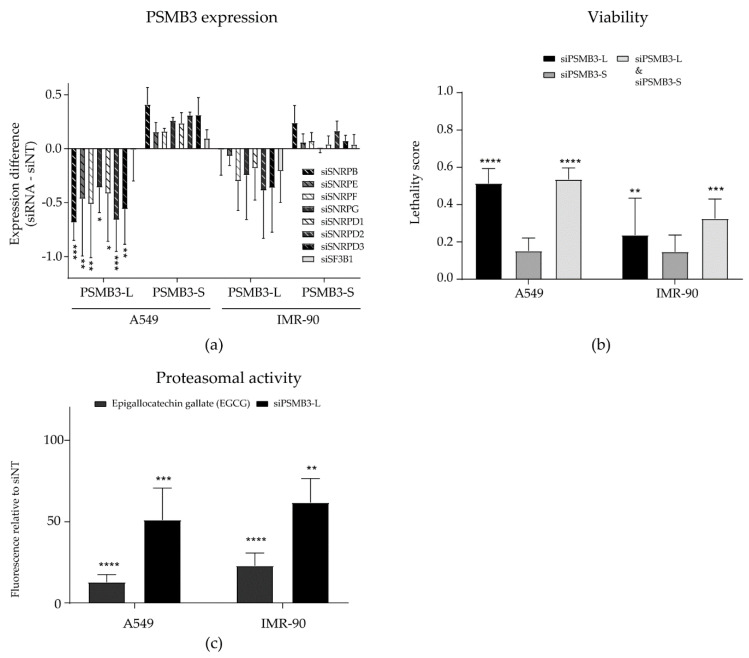
Sm gene silencing induces a cytotoxic alternative splicing switch in PSMB3 more abundantly in non-small cell lung cancer (NSCLC) cells than in non-malignant cells. (**a**) PSMB3 expression was assessed upon silencing all seven Sm genes in A549 NSCLC cells and non-malignant IMR-90 lung fibroblasts. The knockdown of Sm genes led to a significant decrease in PSMB3-L expression in A549 NSCLC cells only. A trend of increased PSMB3-S expression was also observed in these cells, however, this was not significant. In non-malignant IMR-90 lung fibroblasts, a trend of decreased and increased expression of PSMB3-L and PSMB3-S, respectively, was also observed, but this was not significant and to a far lesser extent than observed for A549 NSCLC cells. (**b**) The silencing of PSMB3-L, but not PSMB-S, decreased cell viability in both A549 NSCLC cells and IMR-90 non-malignant lung fibroblasts and was accompanied by decreased proteasomal activity, as measured by N-Succinyl-Leu-Leu-Val-Tyr-7-Amido-4-Methylcoumarin (suc-LLVY-AMC) cleavage (**c**). Error bars indicate the standard deviation. * *p* < 0.05; ** *p* < 0.01; *** *p* < 0.001; **** *p* < 0.0001.

The Sm ring in the core of the spliceosome consists of seven Sm proteins; SmB, SmE, SmF, SmG, SmD1, SmD2 and SmD3. We previously found that all these Sm proteins are cancer-selective lethal targets in NSCLC [10]. Therefore, we investigated whether the alternative splicing of PSMB3 also occurs when the other Sm genes are silenced in A549 NSCLC cells. We observed a significant decrease in PSMB3-L expression when silencing each of the seven Sm genes. Although not significant, this was accompanied by a trend of increased PSMB3-S expression. Again, silencing SF3B1 had no effect on PSMB3 mRNA splicing (Figure 3a).

As silencing Sm genes was lethal to NSCLC cells but not to non-malignant lung cells, we also investigated if PSMB3 splicing was changed after silencing Sm genes in IMR-90 lung fibroblasts. Interestingly, although we observed a slight increase and decrease in PSMB3-S and PSMB3-L expression, respectively, this effect was not significant and occurred to a lesser extent compared to A549 NSCLC cells (Figure 3a). In addition, PSMB3 exon 4 skipping was also not observed when treating either A549 NSCLC cells or IMR-90 non-malignant lung cells with the SF3B1 inhibitor pladienolide B (Figure S4A). Hence, silencing Sm genes, but not the silencing or inhibition of SF3B1, appeared to induce an AS switch in the PSMB3 gene in NSCLC cells only.

We next investigated if the AS switch in PSMB3 compromised cell viability and could thus explain the cancer cell-selective cytotoxicity of Sm gene silencing. To this end, custom siRNAs were designed to specifically target either the PSMB3-L or PSMB3-S variant. The silencing of each PSMB3 variant was confirmed by PCR (Figure S4B). Silencing PSMB3-L induced a slight increase in PSMB3-S expression. Therefore, siRNAs silencing PSMB3-L and PSMB3-S were also combined, to discriminate between the effects caused by the loss of PSMB3-L and the gain of PSMB3-S. Interestingly, silencing PSMB3-L, but not PSMB3-S, resulted in decreased cell viability in both A549 NSCLC cells and IMR-90 fibroblasts (Figure 3b). When co-transfecting cells with both siRNAs, viability was decreased to a similar extent as observed when silencing PSMB3-L alone (Figure 3b). This indicates that the loss of PSMB3-L, and not the increase of PSMB3-S, accounts for the observed cytotoxicity. To investigate if this cytotoxicity could be related to a function of PSMB3 in protein catabolic processes, the proteasomal cleavage of N-Succinyl-Leu-Leu-Val-Tyr-7-Amido-4-Methylcoumarin (Suc-LLVY-AMC) was measured upon silencing PSMB3-L in A549 NSCLC cells (Figure 3c). The proteasome inhibitor epigallocatechin gallate (EGCG) was included as a positive control. PSMB3-L silencing was confirmed with PCR (Figure S4C). Silencing PSMB3-L significantly decreased proteasomal activity, although not as effectively as the proteasome inhibitor, suggesting that the loss of proteasomal activity via the decreased expression of PSMB3-L could contribute to the cytotoxic effect of Sm gene silencing.

**Figure 4:**
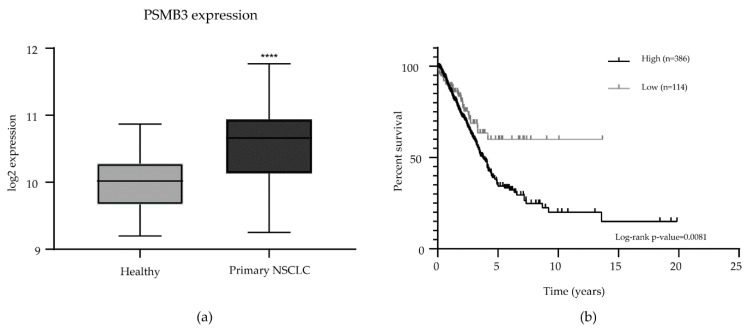
Analysis of publicly available data on PSMB3 expression in clinical materials. (**a**). PSMB3 is overexpressed in NSCLC (*n* = 91) compared to adjacent normal lung tissue samples (*n* = 65) in the NSCLC mixed non-small cell lung carcinoma dataset [14]. Error bars indicate the standard deviation. **** *p* < 0.0001. (**b**). Survival analysis of the Cancer Genome Atlas Lung Adenocarcinoma dataset [15] revealed that high PSMB3 expression corresponds to worse survival in patients with lung adenocarcinomas (log-rank *p*-value < 0.01; high expression *n* = 386; low expression *n* = 114).

Finally, to investigate a possible link between Sm and PSMB3 expression in clinical materials, we analyzed a publicly available gene expression dataset [14] of primary NSCLC samples and normal lung samples. This showed the overexpression of PSMB3 in NSCLC samples (Figure 4a) and revealed a correlation between Sm and PSMB3 transcript expression in both malignant and adjacent normal lung tissues, but not between SF3B1 and PSMB3 expression (Figure S5). Moreover, the analysis of the Cancer Genome Atlas Lung Adenocarcinoma (TCGA-LUAD) dataset [15] revealed that the high expression of PSMB3 is associated with worse survival in lung adenocarcinoma patients (Figure 4b).

## 3. Discussion

There is a growing interest in targeting components of the RNA splicing machinery to treat cancer. A commonly sought therapeutic target in the spliceosome is SF3B1 (reviewed extensively in [16,17,18]). Recently, we identified the core spliceosomal Sm proteins as candidate targets for the selective treatment of NSCLC [10]. In contrast, we found that silencing SF3B1 was toxic for non-malignant lung cells as well [10]. Therefore, in the current study, we set out to compare the transcriptomic effects of silencing SNRPD3 or SF3B1 in NSCLC cells using RNA sequencing. There were notable differences, especially to the extent of DE or AS, where the silencing of SNRPD3 induced substantially fewer DE or AS events than silencing SF3B1. SmD3 is an essential component of the core of the spliceosome. It forms a heptameric ring with six other Sm proteins, together constituting the core of U1, U2, U4 and U5 small nuclear ribonucleoprotein (snRNP) subunits in the spliceosome, encircling the corresponding small nuclear RNA (snRNA). Hence, Sm proteins are present in every step of the RNA splicing reaction. The Sm proteins themselves do not engage in direct interactions with the pre-mRNA, however, the snRNA that they specifically assemble around does so through base pairing with various intronic sequences [19]. In contrast, SF3B1 is part of only the U2 snRNP subunit that participates in every part of the splicing reaction, except the initial step. SF3B1 accommodates the adenosine of the branchpoint sequence in the intronic sequence to support duplex formation with the U2 snRNA [20]. It is unclear whether this difference in the structural versus dynamic functions of SmD3 and SF3B1 can account for the observed difference in the number of DE and AS events upon their silencing. In a study in which the effect of the knockdown of different splicing factors on the AS of 38 selected splicing events was analyzed, the silencing of structural components of the spliceosome (such as Sm proteins) caused more profound changes in AS than silencing transient early components (such as SF3B1) [21]. While this appears to be in contrast to our observations, the authors also found that the knockdown of core splicing factors led to differential effects on specific introns, rather than to inefficient intron splicing in general. In another study, it was found that the depletion of SmB led to AS of cassette exons rather than constitutive exons. Additionally, the knockdown of SmB or SmD1 decreased snRNA expression [22]. It was recently reported that interfering with snRNA levels has a more selective effect on single exons rather than a widespread global effect on splicing efficiency [23]. Together, this suggests that the core of the spliceosome might have a more prominent role in the regulation of specific AS events than might be assumed for a structural component.

When analyzing AS events specific to SNRPD3 silencing compared to SF3B1 silencing, our most prominent finding was a switch in the splicing of PSMB3 mRNA. Specifically, exon 4 was skipped, resulting in a short transcript variant (PSMB3-S) that is predicted to be target for NMD; and thus in decreased PSMB3 expression. This observation was extended to all other Sm genes. Silencing Sm genes, but not SF3B1, generally induced PSMB3 exon 4 skipping in NSCLC cells, but not in non-malignant lung cells. We showed that the loss of the long transcript variant (PSMB3-L) is cytotoxic. We thus identified an AS event that discriminates between the effects of targeting Sm genes versus SF3B1 and that may contribute to the observed cancer-selective lethal phenotype of silencing Sm genes.

The proteasome is a 28-subunit protein complex that degrades ubiquitinated proteins. It consists of a 19S regulatory domain and a 20S core domain. The 20S core domain is built up by two alpha and two beta chains, consisting of subunits α1–7 and β1–7, respectively. PSMB3 codes for the β3 subunit [24]. The β1, β2 and β5 subunits have catalytic activity: caspase-like, trypsin-like and chymotrypsin-like, respectively. In mammalian cells, three types of the 20S proteasome exist: the constitutive proteasome, immunoproteasome and thymoproteasome. Plenty of studies have already shown the relevance of proteasome deregulation in the context of cancer. Cancer cells appear to be specifically sensitive to the inhibition of the constitutive proteasome. Several proteasome inhibitors have been developed and clinically approved as anti-cancer drugs, such as bortezimib, carfilzomib and ixazomib [25]. These proteasome inhibitors mainly target PSMB5.

Interfering with the proteasome has been described to increase the expression of autophagy-related genes, such as LC3-II [26]. However, we did not observe this when we silenced Sm genes in NSCLC cells [10]. Other described downstream effects of proteasome inhibition include the inhibition of the NF-κB pathway, cell cycle arrest through the prevention of p21 degradation and the induction of apoptosis through the activation of the extrinsic and intrinsic pathways [27]. The latter is in line with our previous observation that silencing Sm genes resulted in apoptosis in NSCLC cells [10]. Hence, the inhibition of the proteasome, inducing apoptosis as a direct result of the AS switch in the PSMB3 mRNA, could potentially explain the cytotoxicity of interfering with Sm gene expression in NSCLC cells.

Acquired resistance to, or toxicity caused by, current proteasome inhibiting compounds are problematic [25]. Resistance to these drugs might be overcome by targeting another core 20S subunit; such as PSMB3. Although the PSMB3 subunit is not assumed to have direct proteolytic functions, silencing this subunit was found to contribute to bortezomib sensitivity in human multiple myeloma cells and in A549 NSCLC cells [28]. Of note, in that study, contrasting our observations, cytotoxicity was not evident when silencing PSMB3 alone. We identified a direct link between the core spliceosome and proteasome activity. Although the loss of proteasome activity is clearly not the only response to Sm gene silencing, the lethality of Sm gene silencing and PSMB3 silencing in NSCLC cells was similar, suggesting that the loss of proteasome activity was a major contributor to the selective cytotoxicity of targeting the core spliceosome. Our in vitro observations await in vivo confirmation. In view of the strong cytotoxicity of silencing Sm genes or PSMB3-L in NSCLC cells, such studies will need to be done using an inducible knockdown system.

There are more indications that the proteasome and spliceosome are intertwined. For example, splice factor PRPF19 was shown to interact with PSMB4 of the 20S proteasome, to co-localize with the proteasome after the inhibition of the latter and to exhibit E3 ubiquitin ligase activity [29]. Moreover, in triple-negative breast cancer cells, the expression of proteasomal genes PSMB4 and PSMB5 was dependent on the expression of splice factors PRPF8 and PRPF38A. The treatment of patient-derived triple-negative breast cancer xenografts with the splicing inhibitor E7107 and proteasome inhibitor bortezomib combined showed an enhanced effect compared to either treatment alone [30].

In conclusion, we found that silencing Sm genes indirectly targets the proteasome by inducing a cytotoxic AS switch in PSMB3 mRNA in NSCLC cells only. Potentially, through inhibiting Sm gene expression, the loss of proteasomal activity can be achieved with similar cytotoxic effects as observed when targeting PSMB5, but with more selectivity towards cancer cells. Hence, indirectly targeting the proteasome via the spliceosome might be a powerful strategy in the context of cancer therapy that is worth further investigating.

## 4. Materials and Methods

### 4.1. Cell Culture

Human A549 NSCLC cells (RRID:CVCL_0023) and IMR-90 fetal lung fibroblasts (RRID:CVCL_0347) were purchased from the ATCC (Massanas, VA, USA) and grown in Dulbecco’s modified Eagle’s medium (DMEM, Sigma, part of Merck, Kenilworth, NJ, USA #D5796,) or Eagle’s minimum essential medium (EMEM, Sigma, part of Merck, Kenilworth, NJ, USA #4655), respectively, containing 10% fetal calf serum (PAA Laboratories, part of Thermo Fisher Scientific, Waltham, MA, USA, #A15-101) and 1% penicillin/streptomycin (Sigma, part of Merck, Kenilworth, NJ, USA, #P4333). A549 cell line identity was confirmed by short tandem repeat (STR) analysis (BaseClear, Leiden, The Netherlands) and both cell lines were tested negative for mycoplasma every 3 months. During the experiments, antibiotics were omitted from the medium. All culturing procedures were performed at 37 °C; 5% CO_2_.

### 4.2. siRNA Transfection

A549 and IMR-90 cells were transfected with a 25 nM non-targeting siRNA pool (Dharmacon, part of Horizon Discovery Ltd., Cambridge, UK, siNT, #D-001206-14) or individual siRNAs targeting Sm genes, SF3B1 or PSMB3 (Table S7) and 0.05% DharmaFECT1 (Dharmacon, part of Horizon Discovery Ltd., Cambridge, UK, #T-2001) for A549 or 0.03% Lipofectamine RNAiMAX (Thermo Fisher Scientific, Waltham, MA, USA, #13778) for IMR-90; 24 h after seeding in 96-well or six-well plates. For RNA-seq, two biological replicates were prepared for each sample. All other experiments were performed in triplicate. Cells were harvested 72 h after transfection for RNA isolation or the assessment of proteasomal activity. Cell viability was assessed after 6 days using CellTiter-Blue reagent (Promega, Madison, WI, USA, #G8081). Cell viability was determined by measuring fluorescence at 540 nm excitation and 590 nm emission wavelengths using a Tecan Infinite F200 reader (Tecan Group, Männedorf, Switzerland) and lethality scores [31] were calculated from fluorescent values, as described previously [10].

### 4.3. RNA Isolation, cDNA Preparation and RNA-Seq Library Preparation

Total RNA was isolated from cells using either the miRNeasy Mini Kit (Qiagen, Hilden, Germany, # 217004) with the additional on-column DNAse digestion (RNase-Free DNase Set, Qiagen, Hilden, Germany, # 79254) for RNA-seq experiments, or using phenol–chloroform extraction for PCR experiments using TRIzol reagent (Thermo Fisher Scientific, Waltham, MA, USA, # 15596). The cDNA for PCR experiments was prepared with FIREScript RT cDNA synthesis KIT (Solis Biodyne, Tartu, Estonia, # 06-15-00200). RNA quality for RNA-seq analysis was assessed using the RNA 6000 Nano kit (Agilent, Santa Clara, CA, USA, #5067-1511) on a 2100 Bioanalyzer instrument (Agilent, Santa Clara, CA, USA, # G2939BA) with the 2100 Expert software (RRID:SCR_014466) and determined at a RIN score of 9 or higher. Samples were prepared for RNA-seq using the TruSeq Stranded mRNA Sample Preparation kit (Illumina, San Diego, CA, USA, #RS-122-2101) and Agencourt AMPure XP beads (Beckman Coulter, Brea, CA, USA, #A63880) according to the manufacturer’s protocol using 2 µg input RNA with an extra cleanup step after the PCR amplification of the library to remove potential primer dimers. Sample size and purity was analyzed using the DNA 1000 kit (Agilent, Santa Clara, CA, USA, #5067-1504) on the 2100 Bioanalyzer instrument. Samples were pooled to a final concentration of 10 nM and the pooled library was analyzed using the DNA 7500 kit (Agilent, Santa Clara, CA, USA, #5067-1506). Sequencing was performed on an Illumina HiSeq V4 2500, using a 125 bases paired-end run with an input of 13 pM cDNA.

### 4.4. RNA-Seq Analysis

Sequencing yielded an average of 40 million reads per sample. Raw reads were first subjected to quality control using FastQC version 0.11.4 (RRID:SCR_014583) [32] with default settings and visualized with MultiQC version 0.9 (RRID:SCR_014982) [33] with default parameters. Adapters were trimmed, reads were processed to 120 nt and mapped to the human genome (USCS RefSeq hg19 annotation) using STAR (RRID:SCR_015899) [34]. Differential expression was analyzed using DESeq2 version 1.10 (RRID:SCR_015687) [35] and filtered on logFC < −1 or > 1 and FDR < 0.05. Alternative splicing was detected using the rMATS algorithm version 3.2.5 (RRID:SCR_013049) [13]. Events were filtered on FDR < 0.05 and a threshold for the selection of relevant events was set based on the sum of inclusion and skipping counts calculated by rMATS. Code is available under https://github.com/NKI-TGO/SPLICIFY part 1; the pipeline was developed by Komor et al. [36]. Events with sum counts within the first quartile were excluded (Figure S2; excluding events with total counts < 202 for siSF3B1, < 127 for siSNRPD3). Gene ontology (GO) term analyses were performed in DAVID version 6.8 (RRID:SCR_001881) [11,12].

### 4.5. PCR and Quantitative PCR (qPCR) Experiments

One hundred nanograms of cDNA was added to PCR Master Mix (Thermo Fisher Scientific, Waltham, MA, USA, #K0172), with forward and reverse primers (Table S6), and run on a Biometra T3000 Thermal Cycler (Westburg BV, Utrecht, The Netherlands) using an initial denaturation step at 95 °C for 3 min, followed by 30 cycles of denaturation at 95 °C for 30 s, annealing at 55 °C for 30 s, extension at 72 °C for 1.5 min and a final extension at 72 °C for 7 min. PCR products were analyzed on a 3% agarose gel and band intensity was quantified using ImageJ software version 1.51 (Rasband, W.S., ImageJ, NIH, Bethesda, MD, USA, RRID:SCR_003070). Expression values were normalized to β-actin expression and calculated relative to siNT. Real-time PCR was performed on a LightCycler 480 (Roche) using the HOT FIREPol^®^ EvaGreen^®^ qPCR Mix Plus (no ROX) (Solis BioDyne, Tartu, Estonia, #08-25-00001). Primers were designed using Primer-BLAST 3.0 (RRID:SCR_003095) and purchased as custom oligonucleotides from Invitrogen (Table S6). Expression levels were determined on the basis of the threshold cycle calculated by the LightCycler 480 software (RRID:SCR_012155) and normalized to β-actin. Relative expression levels were calculated compared to siNT (2^−ΔΔCt^).

### 4.6. Proteasome Activity Assay

A549 cells were transfected with siNT or siPSMB3-L, as above. After 3 days, proteasomal activity was quantified using the 20S Proteasome Assay Kit (Cayman Chemical, Ann Arbor, MI, USA, # 10008041) according to the manufacturer’s protocol, measuring fluorescence at 360 nm excitation and 440 nm emission wavelengths on a BioTek plate reader (Agilent). The fluorescent signal relative to siNT-treated cells was calculated.

### 4.7. Expression Data Analysis

The publicly available NSCLC dataset of mixed non-small cell lung carcinoma [14] was analyzed for differential expression between adjacent normal lung tissue samples and primary NSCLC and the correlation between SNRP and PSMB3 expression using R2: Genomics Analysis and Visualization Platform (http://r2.amc.nl) using the built-in one-way ANOVA statistical test to compare between adjacent normal lung tissue samples and primary NSCLC. For survival analysis, TCGA-LUAD data [15] was extracted from the Human Protein Atlas [37].

### 4.8. Statistical Analyses

All experiments were performed in triplicate, except for the RNA sequencing experiments, for which two biological replicates per sample were prepared. For PCR, the viability and proteasome activity experiments’ significant differences compared to siNT or untransfected controls were identified by two-way ANOVA, correcting for multiple comparisons using the Benjamini–Yekutieli method [38]. Correlations were determined through Pearson coefficients. Unless stated otherwise, statistical analyses were performed using GraphPad Prism version 8.2.1 for Windows (GraphPad Software, La Jolla, CA, USA, RRID:SCR_002798).

## Supplementary Materials

The following are available online at https://www.mdpi.com/1422-0067/21/12/4192/s1, Figure S1: Workflow of the RNA sequencing experiment, Figure S2: Histograms depicting the frequency distribution of inclusion and skipping counts calculated by rMATS, Figure S3: PCR or quantitative PCR was performed to validate siSNRPD3-specific AS events predicted by rMATS, Figure S4: Analysis of PSMB3 expression, Figure S5: Analysis of publicly available NSCLC expression dataset, Table S1: DE_siSF3B1, Table S2: DE_siSNRPD3, Table S3: AS_siSNRPD3, Table S4: AS_siSF3B1, Table S5: AS_overlap, Table S6: Primer sequences, Table S7: siRNA sequences.

## Abbreviations

NSCLC	Non-small cell lung cancer
AS	Alternative splicing
