# Supplementary material for: Silencing Core Spliceosome Sm Gene Expression Induces a Cytotoxic Splicing Switch in the Proteasome Subunit Beta 3 mRNA in Non-Small Cell Lung Cancer Cells
Source: Int J Mol Sci. 2020 Jun 12;21(12):4192. doi: 10.3390/ijms21124192 (PMC7349683; doi:10.3390/ijms21124192)
Supplement: Supplementary file 1 [file ijms-21-04192-s001.zip › Supplementary Figures.pdf]

Supplementary Figure 1

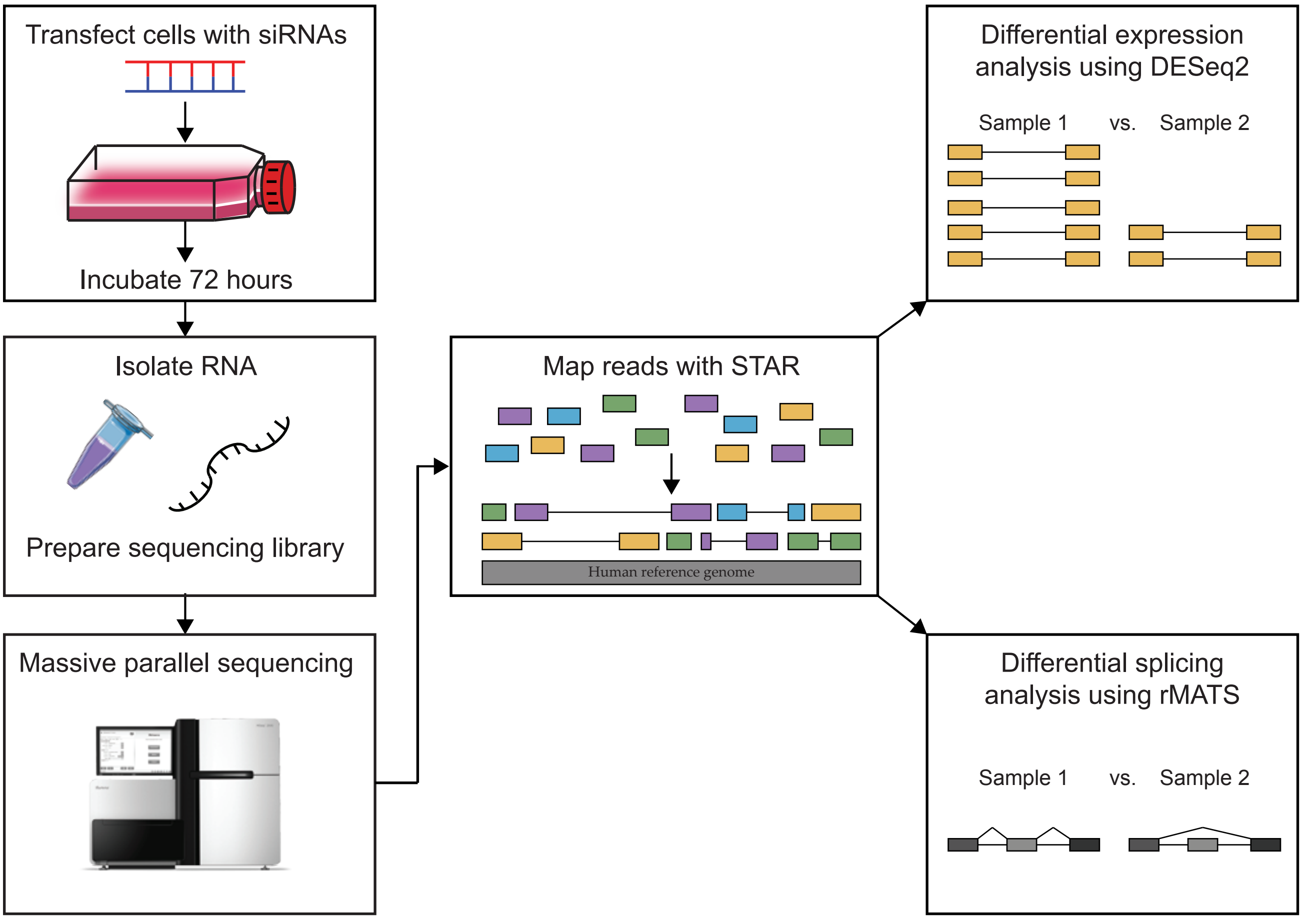

Supplementary Figure 1. Work-flow of the RNA sequencing experiment. A549 NSCLC cells were seeded and transfected with non-targeting siRNA pool (siNT), individual siRNAs targeting either SF3B1 or SNRPD3. Cells were harvested after 72 hours, RNA was isolated, libraries were prepared using the TruSeq Stranded mRNA Sample Preparation kit and sequenced on the Illumina HiSeq V4 2500. Raw reads were subjected to QC, processed and mapped to the human genome (USCS Ref hg19 annotation) using STAR software. Differential expression and alternative splicing patterns were analyzed relative to siNT using DeSeq2 and rMATS, respectively.

Supplementary Figure 2

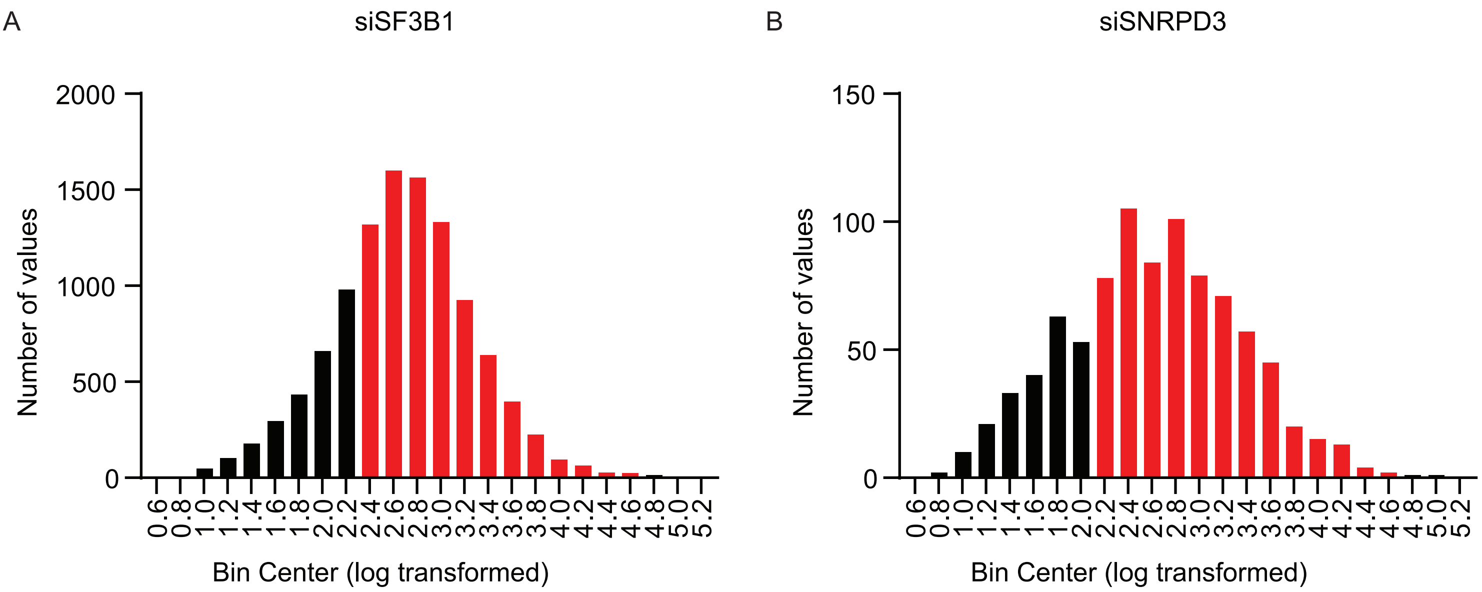

Supplementary Figure 2. Histograms depicting the frequency distribution of inclusion and skipping counts calculated by rMATS for siSF3B1 (A) and siSNRPD3 (B). A treshold was set excluding events with sum counts in the first quartile (in black; < 202 for siSF3B1, < 127 for siSNRPD3).

Supplementary Figure 3

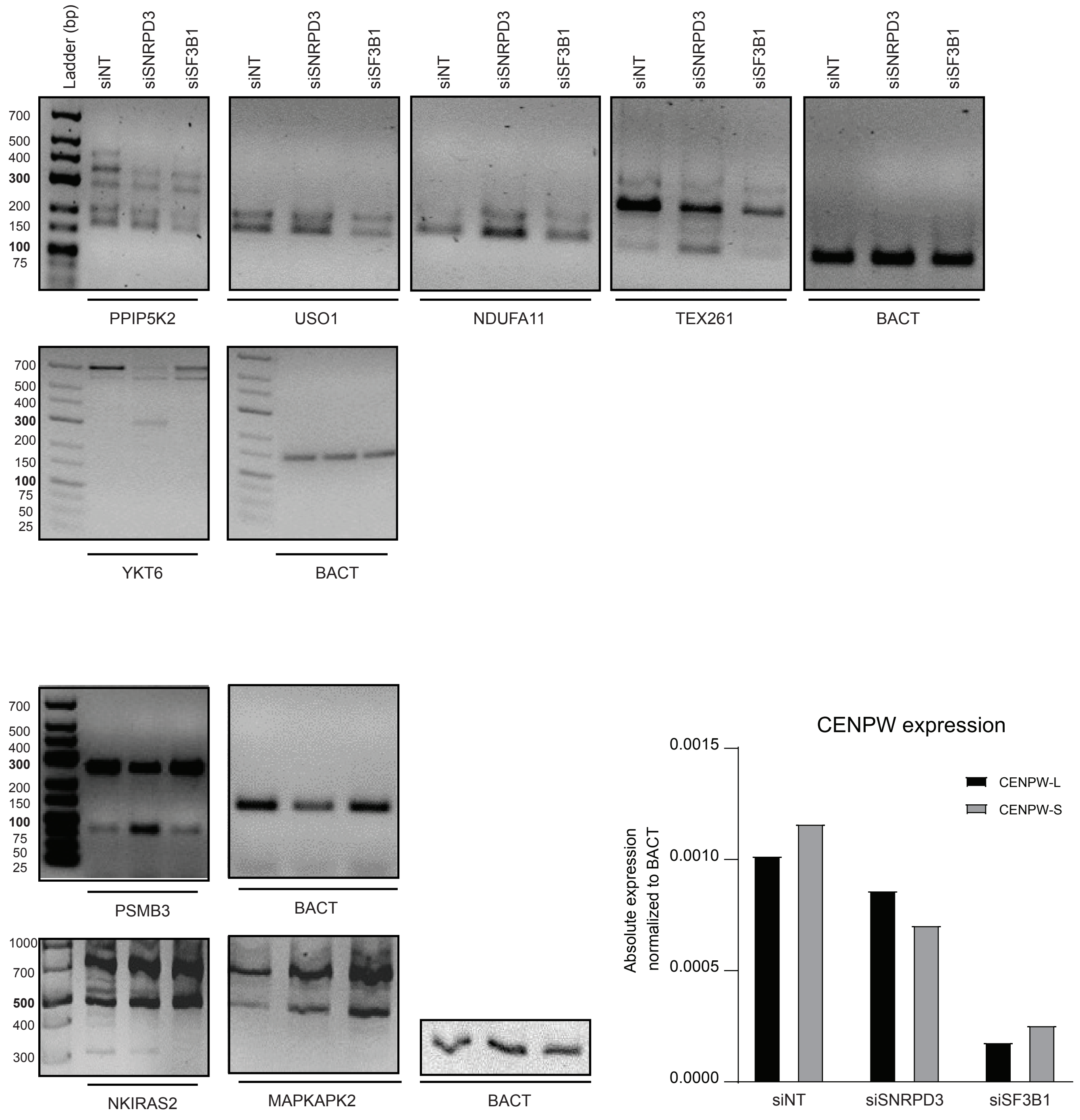

Supplementary Figure 3. PCR or quantitative PCR was performed to validate siSNRPD3-specific AS events predicted by rMATS.

PPIP5K2: exon 28 skipping was predicted. The PCR encompassing exon 27 to exon 31 detects the alternative products observed at 285 bp (exon 28 included) and 165 bp (exon 28 excluded). Indeed, the amount of 285 bp product decreased when silencing SNRPD3, but no increase in the 165 bp product was observed compared to siNT. The other products probably represent the full-length (408 bp); exon 30 skipping (348 bp) and exon 28 plus exon 30 skipping (228 bp) products. These results were not predicted by rMATS and remain rather inconclusive.

USO1: exon 15 retention was predicted. The PCR encompassing exon 14 to exon 16 detects the alternative products observed at 216 bp (exon 15 included) and 195 bp (exon 15 excluded). Both variants are observed in all samples, however, there was no apparent difference between siNT and siSNRPD3 treated cells.

NDUFA11: exon 2 skipping was predicted. The PCR encompassing exon 1 to exon 3 detects the alternative products observed at 276 bp (exon 2 included) and 156 bp (exon 2 excluded). Indeed, in siSNRPD3 treated cells, the short variant appeared to increase in expression, however, this was accompanied by an increase in expression of the long variant as well. Hence, NDUFA11 expression appeared to increase in general after SNRPD3 silencing rather than being alternatively spliced.

TEX261: exon 3 skipping was predicted. The PCR encompassing exon 2 to exon 4 detects the alternative products observed at 254 bp (exon 3 included) and 100 bp (exon 3 excluded). Indeed, the short variant at 100 bp appears to increase when silencing SNRPD3, while the long variant at 254 bp decreases, indicating AS of TEX261. This was not observed when silencing SF3B1. Apart from the predicted products, other bands were observed on the agarose gel that did not match any described or predicted TEX261 splice variants.

YKT6: exon 6 skipping was predicted. The PCR encompassing exon 5 to exon 7 detects the alternative products observed at 717 bp (exon 6 included) and 615 bp (exon 6 excluded). Indeed, the short variant at 615 bp appears to increase when silencing SNRPD3, while the long variant at 717 bp decreases, indicating AS of YKT6. This was also observed when silencing SF3B1, but to a far lesser extent. Moreover, an additional 300 bp product was observed in siSNRPD3 treated cells. This did not match any described or predicted YKT6 splice variants.

PSMB3: exon 4 skipping was predicted. The PCR encompassing exon 3to exon 5 detects the alternative products observed at 258 bp (exon 4 included) and 80 bp (exon 4 excluded). Indeed, the short variant (PSMB3-S) at 80 bp appears to increase when silencing SNRPD3, while the long variant (PSMB3-L) at 258 bp decreases, indicating AS of PSMB3.

NKIRAS2: alternative 5’ splice site usage in exon 3 and exon 3 skipping was predicted. A5’SS usage in exon 3 results in NKIRAS2 transcripts variants with either “full-length” exon 3, a truncated 5’ end of exon 3 (termed exon 3a) or a truncated 3’ end of exon 3 (termed exon 3c). rMATS predicted that the full-length exon 3 was more prevalent in siNT treated cells, whereas the truncated exon 3a was more prevalent in siSNRPD3 treated cells. Finally, exon 3c was predicted to be more skipped in siSNRPD3 treated cells. PCR of exon 2 to exon 4 yields a product at 482 bp (including exon 3) and a product at 314 bp (including exon 3a). These variants are observed, however, there was no apparent difference between siNT and siSNRPD3 treated cells. In siSF3B1 treated cells, the 314 bp product was absent. Additionally, larger products were observed that did not match any described or predicted NKIRAS2 splice variants.

MAPKAPK2: alternative 3’ splice site usage in exon 10 was predicted. This results in a truncated exon 10 in siSNRPD3 treated cells. The PCR encompassing exon 7 to exon 10 should yield products of 1063 bp (full-length exon 10) and 526 bp (truncated exon 10). The truncated variant was indeed detected in all three samples and appeared to increase in both siSNRPD3 and siSF3B1 treated cells. No product was observed at 1063 bp. As the forward primer anneals in exon 7, it is possible that there are cassette exons between exon 7 and exon 10.

CENPW: alternative 3’ splice site usage in exon 1 was predicted. This results in a truncated exon 1 in siNT treated cells, whereas the full-length exon is predicted to be more prevalent in siSNRPD3 treated cells. Primers were designed to specifically recognize either splice variant. Although total CENPW

expression appeared to go down in both siSNRPD3 and siSF3B1 treated cells, the ratio short to long variant indeed increased in siSNRPD3 only.

Supplementary Figure 4

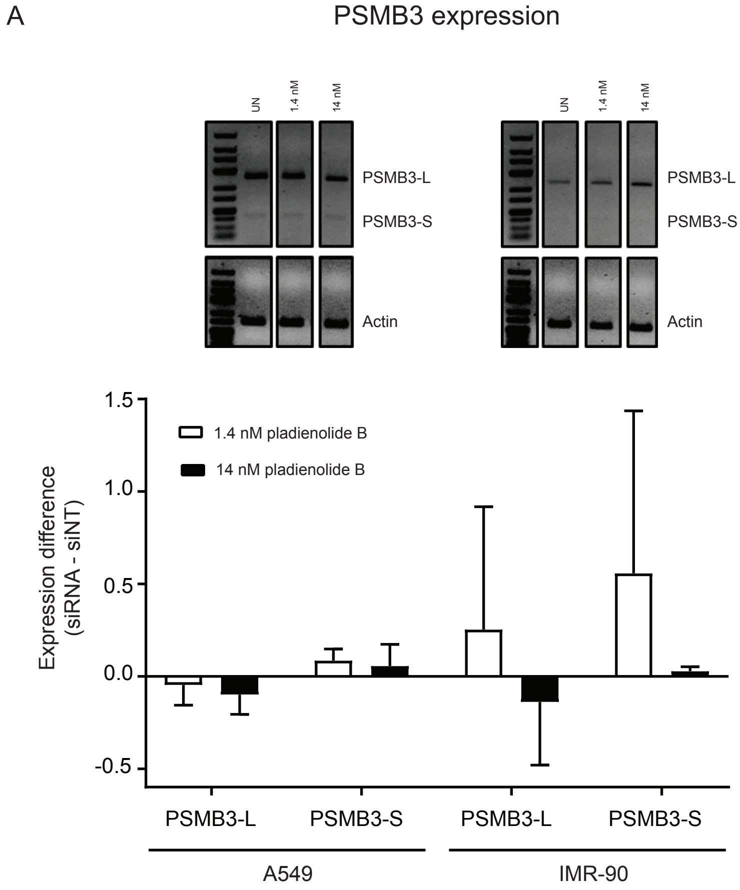

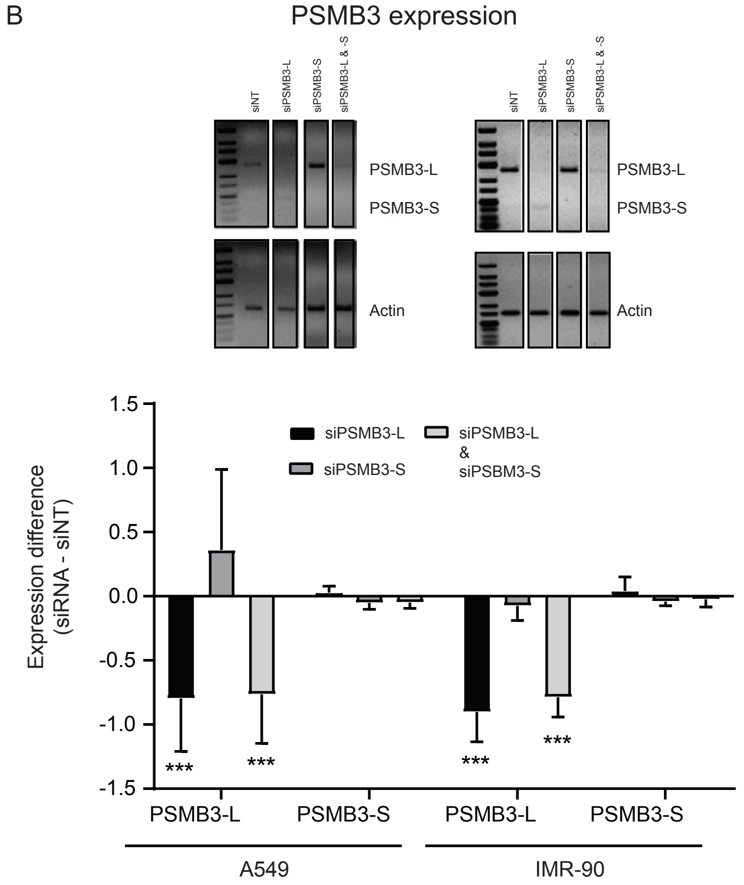

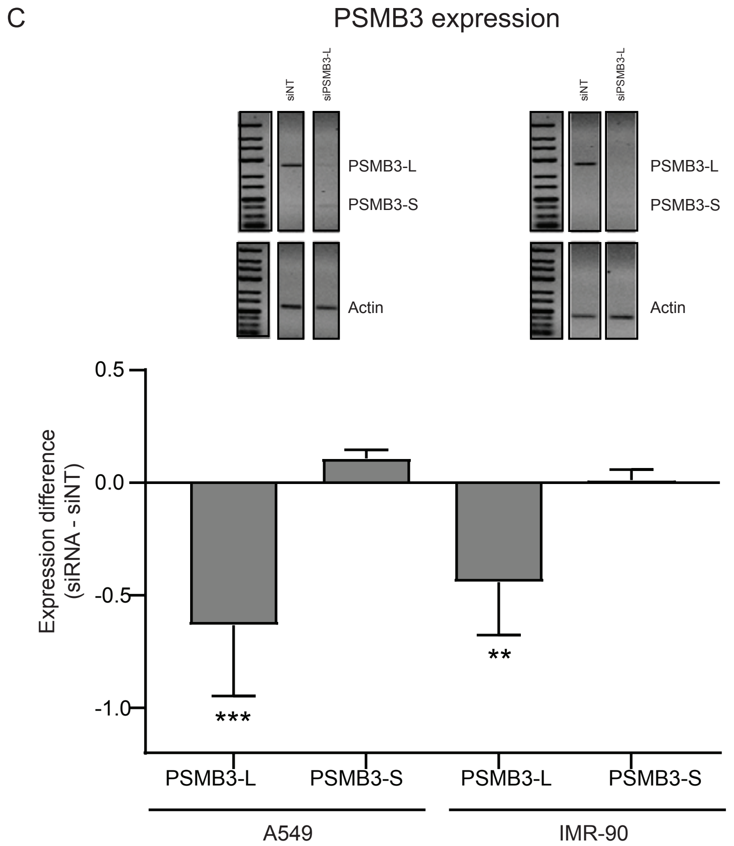

Supplementary Figure 4. Analysis of PSMB3 expression. A. Treatment of A549 and IMR-90 cells with 1.4 nM (which is the IC50 for A549 cells) and 14 nM SF3B1 inhibitor pladienolide B did not induce AS of PSMB3. B and C. Silencing of either PSMB3-L or PSMB3-S was confirmed in samples treated with corresponding siRNAs.

Supplementary Figure 5

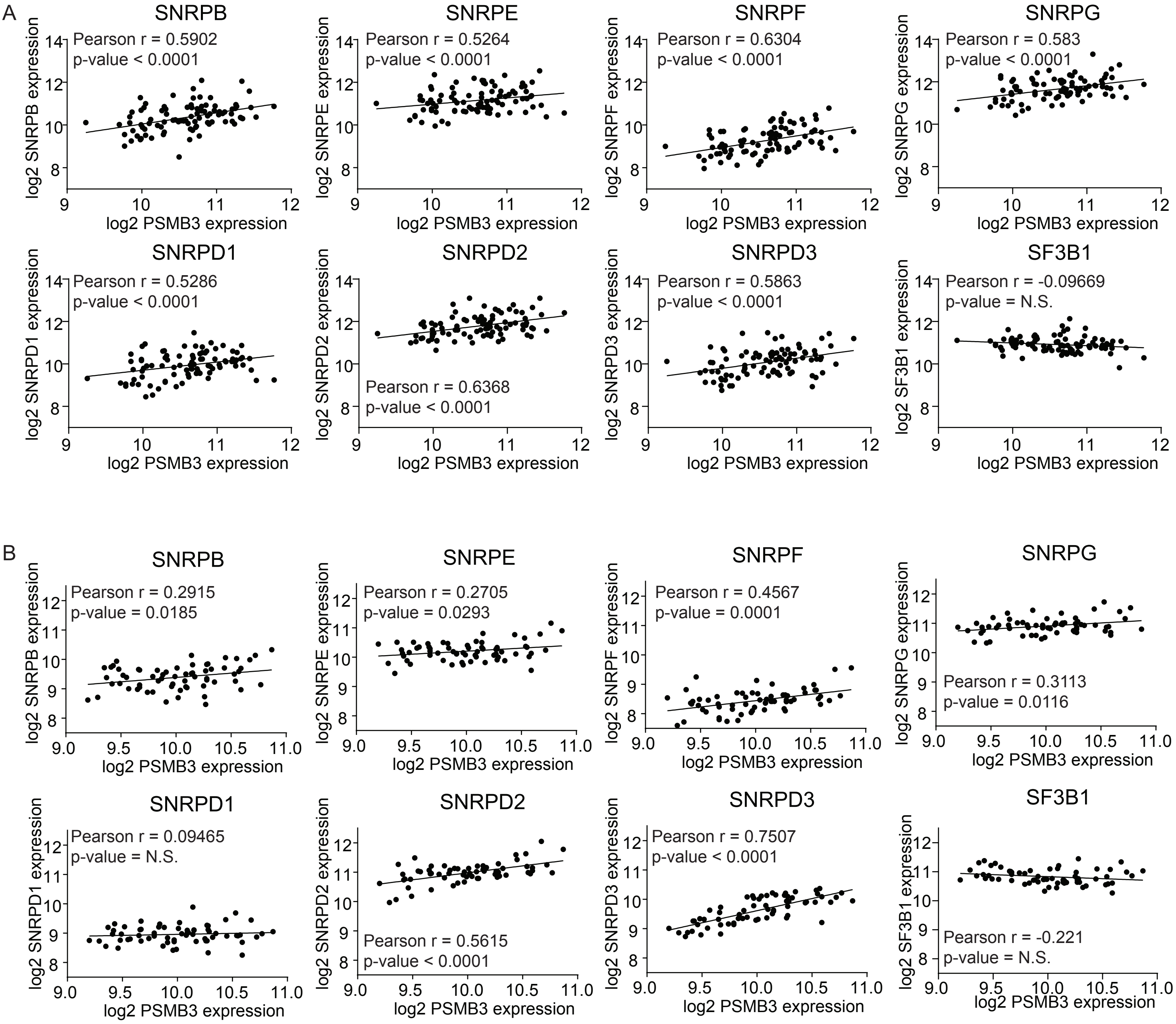

Supplementary Figure 5. Analysis of publicly available NSCLC expression dataset. A significant correlation between Sm protein expression and PSMB3 expression was observed in both NSCLC (A) and healthy (B) lung tissue (except for SNRPD1 in healthy tissue). In contrast, there was no correlation between SF3B1 and PSMB3 expression.
